# Association of Oral Epithelial Dysplasia with Sociodemographic and Clinical Characteristics in Individuals with Oral Lichen Planus and Oral Lichenoid Lesion

**DOI:** 10.4317/jced.63588

**Published:** 2026-02-26

**Authors:** Letícia Côgo Marques, Simone Sant’Anna Gonçalves Barbosa, Danielle Nobre Lopes, Karin Soares Cunha, Arley Silva Junior, Danielle Castex Conde

**Affiliations:** 1 Postgraduate Program in Pathology, School of Medicine, Universidade Federal Fluminense (UFF), Niterói, RJ, Brazil; 2 Technological Innovation Cell, Odontoclínica Central da Marinha, Brazilian Navy, Rio de Janeiro, RJ, Brazil; 3 Department of Pathology, School of Medicine, Hospital Universitário Antônio Pedro, Empresa Brasileira de Serviços Hospitalares (EBSERH), Universidade Federal Fluminense (UFF), Niterói, RJ, Brazil; 4 Hospital Universitário Antônio Pedro, Empresa Brasileira de Serviços Hospitalares EBSERH), Niterói, RJ, Brazil

## Abstract

**Background:**

Oral lichen planus (OLP) and oral lichenoid lesions (OLL) are potentially malignant disorders that share clinical and histopathological features. Oral epithelial dysplasia (OED) is a key indicator of malignant transformation risk in these disorders. This study aimed to investigate possible associations between the severity of OED and sociodemographic variables, hepatitis C infection, and clinical aspects in patients with OLP and OLL.

**Material and Methods:**

The sample comprised 65 biopsies from 56 participants with OLP or OLL, classified according to the criteria of the American Academy of Oral and Maxillofacial Pathology (excluding the absence of OED as a diagnostic criterion for OLP). Data were retrieved from electronic medical records, and photographs taken at the time of biopsy were independently reviewed by two stomatologists and one oral pathologist to classify the clinical patterns. The presence and severity of OED were assessed according to the 2017 World Health Organization system and evaluated independently by two oral pathologists.

**Results:**

A statistically significant association was observed between OED severity and patient age (p=0.004). No significant associations were found with sex, skin color, harmful habits, hepatitis C history, clinical pattern, or anatomical site.

**Conclusions:**

Older patients were more likely to present moderate/severe OED, highlighting the importance of age as a risk factor for progression in OLP and OLL.

## Introduction

Oral lichen planus (OLP) and oral lichenoid lesions (OLL) are inflammatory diseases that share similar clinical and histopathological features (1). Some authors (2 - 5) consider the absence of oral epithelial dysplasia (OED) a diagnostic criterion for classifying OLP; however, the World Health Organization (WHO) (6) classifies it as an oral potentially malignant disorder (OPMD). The presence of OED is considered the gold standard for assessing the risk of malignant transformation in OPMDs (7 - 10), as it reflects underlying genetic and molecular alterations in the oral epithelium (7). Within this context, the aim of the present study was to investigate potential associations between the presence and severity of OED and sociodemographic data, history of hepatitis C, and clinical features of patients with OLP and OLL.

## Material and Methods

This study was approved by the Research Ethics Committee of the Antônio Pedro University Hospital of the Federal Fluminense University (HUAP-UFF), under approval number 5,563,543. It is an observational, cross-sectional, and retrospective study. The sample was obtained through a search of the electronic medical records of the Oral Diagnosis Clinic at HUAP-UFF for patients with OLP, OLL, oral lichenoid mucositis, and oral lichenoid reaction between 2005 and 2019. This search using different terms is justified by the fact that different classification criteria and nomenclatures have been attributed to these lesions over the years. The lesions were reclassified as OLP and OLL according to the clinical and histopathological criteria of the American Academy of Oral and Maxillofacial Pathology (AAOMP), except for the criterion of absence of OED for OLP. OLL due to contact reaction, drug reaction, and asymmetric OLL were considered. The lesions with inadequate histology slides and paraffin blocks for analysis and lesions associated with other OPMDs or diagnosed candidiasis were excluded, based on histopathological analysis through the evaluation of slides stained with hematoxylin and eosin (H&amp;E) and periodic acid-Schiff (PAS). These cases were excluded to exclusively evaluate OLP and OLL and because the epithelial cells altered by candidiasis can also exhibit inflammatory architectural and cytological changes similar to OED, complicating the evaluation (11). The data collected from the electronic medical records included gender, age, skin color, history of hepatitis C, and harmful habits (smoking and drinking), and the anatomical site of the biopsies. For the classification of clinical patterns (reticular/papular, atrophic, erosive/ulcerative, plaque, and vesicular), photographs from the day of the biopsy contained in the electronic medical records were re-evaluated independently by two stomatologists (ASJ and LCM) and an oral pathologist with experience in stomatology (DCC), and discordant cases were discussed together. The reticular pattern was considered in the presence of fine white linear or lace-like lines (Wickham's striae); the plaque pattern in the presence of homogeneous white plaques; the atrophic pattern in the presence of erythematous areas with white striations on the margins; the erosive/ulcerative pattern in the presence of erosive and ulcerative lesions covered by a yellowish-white pseudomembrane, with white striations on the margins; and the vesicular/bullous pattern by the presence of fluid-filled vesicles or blisters, which may rupture to form erosions. For the analysis the presence and degree of OED, all sections of the histological slides were reviewed by two experienced oral pathologists (DCC and KSC), independently, to evaluate the absence or presence of OED and its severity level, according to the 2017 WHO criteria (12). Discordant data were discussed together. The sections were analyzed entirely; however, the margins of the lesions were not considered to avoid artifacts that could interfere with the analysis. The assessment was performed blinded to the original histopathological evaluation and clinical data of the participants. To identify the presence or absence of OED, the cytological and architectural criteria recommended by the WHO were observed (12). Regarding the degree of OED, mild OED was defined when these architectural and cytological changes were restricted to the lower third of the epithelium; moderate OED was defined when such changes extended to the middle third; and severe or intense OED was defined when the changes affected the upper third of the epithelium (12). For the evaluation of associations between OED and the other variables, lesions were regrouped into two categories according to the WHO grading system: Group 1, comprising biopsies with no or mild OED, and Group 2, comprising biopsies with moderate and severe OED (10 , 13 , 14). The Statistical Package for the Social Sciences® (SPSS), version 23.0, was used for the analyses and the level of significance was set at 5% (p 0.05). Qualitative variables were presented as relative frequency and absolute frequency [%(n)], while quantitative variables were presented as mean (standard deviation) and median (minimum-maximum). The analysis of the normality of the quantitative variables was conducted by graphical method and specific statistical tests (Shapiro-Wilk and Kolmogorov-Smirnov). For the comparison between the two studied groups, the Chi-squared test or Fisher's exact test was used for qualitative variables, with their respective measures of association (Cramer's V and Phi, respectively). The student's t-test for independent samples or the Mann-Whitney test was used for quantitative variables, depending on the result of the normality test.

## Results

The sample consisted of 65 biopsies (54 from OLP and 11 from OLL) from 56 participants. Seven participants had more than one biopsy from different areas and/or taken at different times. Among the participants diagnosed with OLL, four (36.4%) were classified as having contact reaction OLL; four (36.4%) as drug-induced OLL; and three (27.2%) as asymmetric OLL. The sociodemographic data, history of hepatitis C, and clinical aspects of the participants according to the diagnosis (OLP and OLL) are shown in Table 1.

Table 1

The participants' ages ranged from 28 to 93 years, with a mean age of 57.6 (±13.6) years for OLP and 53.4 (±12.3) years for OLL. Most participants were female (n=42/75.0%), white (n=32/57.1%), and without harmful habits such as smoking and drinking (n=20/35.7%). The presence of hepatitis C virus infection was identified in 10.9% (n=5) of the participants with OLP. The majority of lesions were located on the buccal mucosa (n=39/60.0%) and the tongue (n=15/23.1%). The most frequent clinical patterns were reticular (n=18/27.7%) and plaque (n=17/26.2%). Seven (10.8%) slides came from lesions that lacked photographic data and records in the medical file about the exact region of the biopsy, making it impossible to classify their patterns (Table 1). According to WHO criteria, 18 (27.7%) showed no OED, 35 (53.8%) were classified as mild OED, 12 (18.5%) as moderate OED, and none as severe OED. Most participants with OLP who showed OED were over 50 years old (n=18/28 mild OED, 64.3%; and n=10/11 moderate OED, 90.9%) and were white (n=18/28 mild OED, 64.3%; and n=9/11 moderate OED, 81.8%). Biopsies from participants who were both smokers and alcohol consumers showed a higher frequency of OED (n=9/15, 60.0%). Among participants with a history of hepatitis C, most biopsies presented OED (n=6/8 with mild OED; and n=1/8 with moderate OED). No participant with OLP presented severe OED. (Table 2).

Table 2

Most of the OLP lesions that exhibited moderate OED were located on the buccal mucosa (n=7; 53.8%) and on the tongue (n=2; 15.4%) and presented reticular (n=5; 38.5%), plaque (n=2; 15.4%), and atrophic patterns (n=2;15.4%) (Table 2). Among participants with OLL, none presented severe OED. One case of moderate OED (9.1%) was identified in a female, white, non-smoker, and hepatitis C-negative patient (Table 2). Regarding OLL, of the four biopsies diagnosed as contact reaction OLL, one (25.0%) showed no OED, two (50.0%) mild OED, and one (25.0%) moderate OED. Of three biopsies with asymmetric OLL, one (33.3%) showed no OED and two (66.6%) moderate OED. Of the four drug-induced OLL, one (25.0%) showed no OED and three (75.0%) mild OED. Statistically significant associations were found between age and the degree of OED (t=-2.994; df=63; p=0.004) (Table 3).

Table 3

Participants with no/mild OED (n=52) had a mean age of 55.3 ± 12.8 years, whereas those with moderate/severe OED (n=12) had a mean age of 67.6 ± 13.1 years, with a mean difference of -12.34 (95% CI: -20.57 to -4.10). No significant associations were found between OED severity and gender, harmful habits, history of hepatitis C, anatomical site, or clinical pattern of the lesions (Table 3).

## Discussion

This study provides a comprehensive analysis of OED in OLP and OLL by exploring not only its prevalence and severity, but also its relationship with sociodemographic and clinical variables, including hepatitis C history. The results showed a statistically significant association between the degree of OED and the age (p=0.004). Older individuals were more likely to have moderate/severe OED than younger individuals. These findings are noteworthy, considering that genetic mutations accumulate in epithelial tissues over time, contributing to the development of OED (15). Such findings are of significant importance, as moderate to severe OED is associated with an increased risk of progression to oral cancer (10). Therefore, it is crucial for oral healthcare professionals, particularly dentists, to be aware of the need to monitor patients with OLP and OLL, especially those with this sociodemographic profile. According to the literature, the risk of malignant transformation of OLP and OLL is greater in lesions located on the tongue, in the presence of erosive and/or atrophic areas, and when associated with consumption of tobacco and/or alcohol and infection with the hepatitis C virus (16 - 18). However, we did not find a statistically significant difference regarding the degree of OED with variables such as sex, vicious habits (smoking and alcoholism), history of hepatitis C, clinical pattern, and anatomical site of the lesions. In comparison with other DPMs, a study by Azevedo et al. (14), in 2021, involving lesions of leukoplakia, erythroplakia, leukoerythroplakia, and actinic cheilitis, found a statistically significant association between increased age (p=0.048), alcohol use (p=0.032), and the presence of ulceration (p&lt;0.0001) with an increased degree of OED. Additionally, lesions located on the ventral surface of the tongue, buccal mucosa, floor of the mouth, lateral border of the tongue, lower lip, hard palate, and soft palate showed a higher frequency of moderate and severe OED (p&lt;0.0001). However, these authors did not find a statistically significant association between gender (p=0.609) and tobacco use (p=0.438) with an increased degree of OED (14). Regarding the clinical pattern variable, the retrospective design of the study, combined with our clinic's protocols-recommending biopsies from representative areas and allowing multiple patterns per sample (4)-may have limited the individual assessment of these patterns. Additionally, OED caused by reactive/inflammatory atypia could have contributed to the distortion of the analysis of the association with some variables. Although most traditional risk factors such as smoking, alcohol consumption, hepatitis C infection, and clinical presentation did not show significant associations with OED severity in this study, the strong correlation with age highlights the importance of close monitoring of elderly patients. This has practical implications for clinical follow-up and reinforces the need for long-term surveillance strategies in routine dental practice. In conclusion, patient age is a significant risk factor for the development of moderate/severe OED in patients with OLP and OLL. Older individuals were more likely to have moderate/severe OED than younger individuals. These findings reinforce the need for vigilance in the clinical management of older individuals with OLP and OLL, even in the absence of traditional risk factors, since early detection of OED is essential to prevent malignant transformation.

## Figures and Tables

**Table 1:** Sociodemographic data, history of hepatitis C and lesions clinical aspects of the participants.

Characteristics	OLP(n=46; 82.1%)		OLL(n=10; 17.9%)		Total(n=56; 100.0%)	
Age						
Mean (sd) years	57.6 (±13.6)		53.4 (±12.3)		56.9 (±13.3)	
Median	59.0		52.0		58.0	
Minimum	28		31		28	
Maximum	93		66		93	
i.r	16.0		16.8		16.5	
< 50 years	14	30.4%	4	40.0%	18	32.1%
≥ 50 years	32	69.6%	6	60.0%	38	67.9%
Sex						
Female	35	76.1%	7	70.0%	42	75.0%
Male	11	23.9%	3	30.0%	14	25.0%
Skin Color						
White	30	65.2%	2	20.0%	32	57.1%
Black	16	34.8%	8	80.0%	24	42.9%
Harmful habits						
Absent	17	37.0%	3	30.0%	20	35.7%
Smoker	8	17.4%	2	20.0%	10	17.9%
Alcoholic	6	13.0%	0	0.0%	6	10.7%
Smoker and alcoholic	12	26.1%	5	50.0%	17	30.4%
No information	3	6.5%	0	0.0%	3	5.4%
Hepatitis C						
Yes	5	10.9%	0	0.0%	5	8.9%
No	37	80.4%	9	90.0%	46	82.1%
No information	4	8.7%	1	10.0%	5	8.9%
Anatomical Sites						
Buccal mucosa	35	64.8%	4	36.4%	39	60.0%
Tongue	9	16.7%	6	54.5%	15	23.1%
Gingiva/alveolar ridge	4	7.4%	0	0.0%	4	6.2%
Lips	3	5.6%	0	0.0%	3	4.6%
Palate	2	3.7%	0	0.0%	2	3.1%
Vestibule	1	1.9%	0	0.0%	1	1.5%
Labial mucosa	0	0.0%	1	9.1%	1	1.5%
Clinical Pattern						
R	18	33.3%	0	0.0%	18	27.7%
P	11	20.4%	5	45.5%	16	24.6%
R+P	7	13.0%	3	27.3%	10	15.4%
A	4	7.4%	0	0.0%	4	6.2%
E/U	3	5.6%	0	0.0%	3	4.6%
A+P	2	3.7%	1	9.1%	3	4.6%
R+P+A	1	1.9%	1	9.1%	2	3.1%
R+A	1	1.9%	0	0.0%	1	1.5%
E/U+P	0	0.0%	1	9.1%	1	1.5%
NC	7	13.0%	0	0.0%	7	10.8%

s.d.= standard deviation; i.r = Interquartile range. Combinations of clinical patterns were found in the biopsy areas. R=Reticular; A=Atrophic; E/U= Erosive/Ulcerative; P=Plaque; NC=No classification. Lesions from biopsies of the 56 participants were considered. Seven (six from LPO and one from LLO) participants had more than one biopsy from different areas and/or taken at different times.

**Table 2:** Distribution of the degree of epithelial dysplasia with sociodemographic data, history of hepatitis C and clinical aspects in participants with oral lichen planus and oral lichenoid lesion.

	OLP (n=54; 83.1%)										OLL (n=11; 16.1%)						
	No(n=15;27.8%)		Mild(n=28; 51.9%)		Moderate (n=11; 20.4%)		Severe(n=0;0.0%)		No(n=3;27.3%)		Mild(n=7;63.6%)		Moderate (n=1;9.1%)		Severe(n=0;0.0%)		
Age																	
< 50 years (n=15 and n=5)	4	7.4%	10	18.5%	1	1.9%	0	0.0%	2	18.2%	3	27.3%	0	0.0%		0	0.0%
≥ 50 years (n=39 and n=6)	11	20.4%	18	33.3%	10	18.5 %	0	0.0%	1	9.1%	4	36.4%	1	9.1%		0	0.0%
Sex																	
Female (n=40 and n=7)	10	18.5%	23	42.6%	7	13.0%	0	0.0%	3	27.3%	3	27.3%	1	9.1%		0	0.0%
Male (n=14 and n=4)	5	9.3%	5	9.3%	4	7.4%	0	0.0%	0	0.0%	4	36.4%	0	0.0%		0	0.0%
Skin color																	
White (n=37 and n=2)	10	18.5%	18	33.3%	9	16.7%	0	0.0%	0	0.0%	1	9.1%	1	9.1%		0	0.0%
Black (n=17 and n=9)	5	9.3%	10	18.5%	2	3.7%	0	0.0%	3	27.3%	6	54.5%	0	0.0%		0	0.0%
Harmful habits																	
Absent (n=18 and n=3)	3	5.6%	10	18.5%	5	9.3%	0	0.0%	0	0.0%	2	18.2%	1	9.1%		0	0.0%
Smokers (n=9 and n=2)	3	5.6%	4	7.4%	2	3.7%	0	0.0%	2	18.2%	0	0.0%	0	0.0%		0	0.0%
Alcoholics (n=6 and n=0)	1	1.9%	5	9.3%	0	0.0%	0	0.0%	0	0.0%	0	0.0%	0	0.0%		0	0.0%
Smokers and Alcoholics (n=15 and n=6)	6	11.1%	5	9.3%	4	7.4%	0	0.0%	1	9.1%	5	45.5%	0	0.0%		0	0.0%
No information (n=6 and n=0)	2	3.7%	4	7.4%	0	0.0%	0	0.0%	0	0.0%	0	0.0%	0	0.0%		0	0.0%
Hepatitis C																	
Yes (n=8 and n=0)	1	1.9%	6	11.1%	1	1.9%	0	0.0%	0	0.0%	0	0.0%	0	0.0%		0	0.0%
No (n=42 and n=10)	11	20.4%	21	38.9%	10	18.5%	0	0.0%	2	18.2%	7	63.6%	1	9.1%		0	0.0%
No information (n=4 and n=1)	3	5.6%	1	1.9%	0	0.0%	0	0.0%	1	9.1%	0	0.0%	0	0.0%		0	0.0%
Anatomical sites																	
Buccal mucosa (n=35 and n=4)	10	18.5%	18	33.3%	7	13.0%	0	0.0%	2	18.2%	2	18.2%	0	0.0%		0	0.0%
Tongue (n=9 and n=6)	2	3.7%	5	9.3%	2	3.7%	0	0.0%	0	0.0%	5	45.5%	1	9.1%		0	0.0%
Gingiva/ Alveolar ridge (n=4 and n=0)	3	5.6%	0	0.0%	1	1.9%	0	0.0%	0	0.0%	0	0.0%	0	0.0%		0	0.0%
Lips (n=3 and n=0)	0	0.0%	3	5.6%	0	0.0%	0	0.0%	0	0.0%	0	0.0%	0	0.0%		0	0.0%
Palate (n=2 and n=0)	0	0.0%	2	3.7%	0	0.0%	0	0.0%	0	0.0%	0	0.0%	0	0.0%		0	0.0%
Vestibule (n=1 and n=0)	0	0.0%	0	0.0%	1	1.9%	0	0.0%	0	0.0%	0	0.0%	0	0.0%		0	0.0%
Labial mucosa (n=0 and n=1)	0	0.0%	0	0.0%	0	0.0%	0	0.0%	1	9.1%	0	0.0%	0	0.0%		0	0.0%
Clinical Pattern																	
R (n=18 and n=0)	6	11.1%	9	16.7%	3	5.6%	0	0.0%	0	0.0%	0	0.0%	0	0.0%		0	0.0%
P (n=11 and n=5)	3	5.6%	6	11.1%	2	3.7%	0	0.0%	2	18.2%	3	27.3%	0	0.0%		0	0.0%
R+P (n=7 and n=3)	3	5.6%	3	5.6%	1	1.9%	0	0.0%	1	9.1%	2	18.2%	1	0.0%		0	0.0%
A (n=4 and n=0)	1	1.9%	2	3.7%	1	1.9%	0	0.0%	0	0.0%	0	0.0%	0	0.0%		0	0.0%
E/U (n=3 and n=0)	0	0.0%	2	3.7%	1	1.9%	0	0.0%	0	0.0%	0	0.0%	0	0.0%		0	0.0%
A+P (n=2 and n=1)	1	1.9%	0	0.0%	1	1.9%	0	0.0%	0	0.0%	1	9.1%	0	0.0%		0	0.0%
R+P+A (n=1 and n=1)	0	0.0%	1	1.9%	0	0.0%	0	0.0%	0	0.0%	0	0.0%	1	9.1%		0	0.0%
R+A (n=1 and n=0)	0	0.0%	1	1.9%	0	0.0%	0	0.0%	0	0.0%	0	0.0%	0	0.0%		0	0.0%
E/U+P (n=0 and n=1)	0	0.0%	0	0.0%	0	0.0%	0	0.0%	0	0.0%	1	9.1%	0	0.0%		0	0.0%
NC (n=7 and n=0)	1	1.9%	4	7.4%	2	3.7%	0	0.0%	0	0.0%	0	0.0%	0	0.0%		0	0.0%

Of the 56 participants (46 with OLP and ten with OLL), seven (six with OLP and one with OLL) had more than one biopsy from different areas and/or performed at different times. Participants’ ages at the time of biopsy were considered. Combinations of clinical patterns were found in the biopsy areas. R=Reticular; A=Atrophic; E/U= Erosive/Ulcerative; P=Plaque; NC=no classification.

**Table 3:** Association of the degree of epithelial dysplasia with sociodemographic data, history of hepatitis C and clinical aspects in participants with oral lichen planus and oral lichenoid lesion.

	Total					
	Mild(n=53; 81,5%)		Moderate/Severe(n=11; 18.5%)		p*	Î¦
Age					0.004¥	-
< 50 years (n=20)	19	29.2%	1	1.5%		
≥ 50 years (n=45)	34	52.3%	11	16.9%		
Sex					0.724§	0.060
Female (n=47)	39	60.0%	8	12.3%		
Male (n=18)	13	21.5%	4	6.2%		
Skin Color						
White (n=39)	29	44.6%	10	15.4%	0.103§	-0.227
Black (n=26)	24	36.9%	2	3.1%		
Harmful habits						
Absent (n=21)	15	23.1%	6	9.2%	0.315§	0.152
Smokers (n=11)	9	13.8%	2	3.1%	1.000§	-0.026
Alcoholics (n=6)	6	9.2%	0	0.0%	0.330§	-0.170
Smokers and alcoholics (n=21)	17	26.2%	4	6.2%	1.000§	-0.024
No informations (n=6)	6	9.2%	0	0.0%		
Hepatitis C					1.000§	-0.074
Yes (n=8)	7	10.8%	1	1.5%		
No (n=52)	41	63.1%	11	16.9%		
No informations (n=5)	5	7.7%	0	0.0%		
Anatomical sites						
Buccal mucosa (n=39)	32	49.2%	7	10.8%	1.000§	-0.016
Tongue (n=15)	12	18.5%	3	4.6%	1.000§	0.022
Gingiva/alveolar ridge (n=4)	3	4.6%	1	1.5%	0.576§	0.043
Lips (n=3)	3	4.6%	0	0.0%	1.000§	-0.105
Palate (n=2)	2	3.1%	0	0.0%	1.000§	-0.085
Vestibule (n=1)	0	0.0%	1	1.5%	0.185§	0.263
Labial mucosa (n=1)	1	1.5%	0	0.0%	1.000§	-0.059
Clinical pattern						
Reticular (n=31)	26	40.0%	5	7.7%	1.000§	-0.032
Plaque (n=32)	27	41.5%	5	7.7%	0.740§	-0.047
Atrofic (n=10)	7	10.8%	3	4.6%	0.353§	0.154
Erosive/Ulcerative (n=4)	3	4.6%	1	1.5%	0.541§	0.056
No information (n=7)	5	7.7%	2	3.1%	-	-

Of the 56 participants (46 with OLP and ten with OLL), seven (six with OLP and one with OLL) had more than one biopsy from different areas and/or performed at different times. Participants’ ages at the time of biopsy were considered. The combination of moderate and severe epithelial dysplasia categories was performed, considering that more than 20% of the number of the cells were fewer than 5 and due to the low clinical impact. More than one pattern was observed in the same participant in most cases. *p (p≤0.05); ¥ Student’s t test; §Fisher’s exact test; Φ Phi coefficient.
